# TNFα-mediated necroptosis in brain endothelial cells as a potential mechanism of increased seizure susceptibility in mice following systemic inflammation

**DOI:** 10.1186/s12974-022-02406-0

**Published:** 2022-02-02

**Authors:** Wan-Yu Huang, Yen-Ling Lai, Ko-Hung Liu, Shankung Lin, Hsuan-Ying Chen, Chih-Hung Liang, Hung-Ming Wu, Kuei-Sen Hsu

**Affiliations:** 1 Institute of Basic Medical Sciences Basic Medicine, College of Medicine, National Cheng-Kung University, Tainan, Taiwan; 2 Pediatrics of Kung-Ten General Hospital, Taichung City, Taiwan; 3 Inflammation Research and Drug Development Center, Changhua Christian Hospital, Changhua, Taiwan; 4 Department of Food Science, Tunghai University, Taichung City, Taiwan; 5 Department of Neurology, Changhua Christian Hospital, Changhua City, Taiwan; 6 Institute of Acupuncture, School of Chinese Medicine, China Medical University, Taichung City, Taiwan

**Keywords:** Systemic inflammation, Sepsis, Seizure susceptibility, Kainic acid, Necroptosis, Astrocytic Kir4.1, Endothelia, Blood–brain barrier, Vascular integrity

## Abstract

**Background:**

Systemic inflammation is a potent contributor to increased seizure susceptibility. However, information regarding the effects of systemic inflammation on cerebral vascular integrity that influence neuron excitability is scarce. Necroptosis is closely associated with inflammation in various neurological diseases. In this study, necroptosis was hypothesized to be involved in the mechanism underlying sepsis-associated neuronal excitability in the cerebrovascular components (e.g., endothelia cells).

**Methods:**

Lipopolysaccharide (LPS) was used to induce systemic inflammation. Kainic acid intraperitoneal injection was used to measure the susceptibility of the mice to seizure. The pharmacological inhibitors C87 and GSK872 were used to block the signaling of TNFα receptors and necroptosis. In order to determine the features of the sepsis-associated response in the cerebral vasculature and CNS, brain tissues of mice were obtained for assays of the necroptosis-related protein expression, and for immunofluorescence staining to identify morphological changes in the endothelia and glia. In addition, microdialysis assay was used to assess the changes in extracellular potassium and glutamate levels in the brain.

**Results:**

Some noteworthy findings, such as increased seizure susceptibility and brain endothelial necroptosis, Kir4.1 dysfunction, and microglia activation were observed in mice following LPS injection. C87 treatment, a TNFα receptor inhibitor, showed considerable attenuation of increased kainic acid-induced seizure susceptibility, endothelial cell necroptosis, microglia activation and restoration of Kir4.1 protein expression in LPS-treated mice. Treatment with GSK872, a RIP3 inhibitor, such as C87, showed similar effects on these changes following LPS injection.

**Conclusions:**

The findings of this study showed that TNFα-mediated necroptosis induced cerebrovascular endothelial damage, neuroinflammation and astrocyte Kir4.1 dysregulation, which may coalesce to contribute to the increased seizure susceptibility in LPS-treated mice. Pharmacologic inhibition targeting this necroptosis pathway may provide a promising therapeutic approach to the reduction of sepsis-associated brain endothelia cell injury, astrocyte ion channel dysfunction, and subsequent neuronal excitability.

**Supplementary Information:**

The online version contains supplementary material available at 10.1186/s12974-022-02406-0.

## Introduction

Seizure is a common acute complication of sepsis and systemic inflammation [1–3]. It is induced by the hyperexcitability of circuits, caused by an imbalance between neuronal excitatory and inhibitory activities in the brain. Sepsis resulting in the excessive production of proinflammatory factors in the peripheral circulation and brain is believed to be a crucial factor in the pathogenesis of neuronal hyperexcitation, leading to an enhanced predisposition to seizure, and subsequent changes in neuroplasticity, which may evolve into a chronic seizure syndrome [3–6]. Our previous study showed that a single intraperitoneal (i.p.) injection of lipopolysaccharide (LPS) in mice increases their susceptibility to pentylenetetrazole-induced seizures [7]. Similar observations have been noted in postnatal and adult animals following systemic inflammation [3, 6, 8]. This evidence indicates that inflammatory processes initially from the peripheral circulation to the brain are a common and crucial mechanism in the pathophysiology of seizures and epilepsy. However, how unbalanced regulation of systemic inflammation contributes to seizure development is still unclear.

The blood–brain barrier (BBB) is a major part of the interface between the peripheral circulation and the central nervous systems (CNS). The BBB is involved in managing the exchange of materials between the blood and the brain in maintaining a stable CNS microenvironment. It has long been proposed that dysfunction of the BBB might contribute to the development of epilepsy and epileptogenesis [3, 9, 10]. The BBB is composed of endothelial cells, tight junctions, basal lamina, and associated cells, including astrocytic end-feet and pericytes. The continuous non-fenestrated endothelial cells form a tight monolayer as endothelial barrier, which plays a central role in maintaining BBB integrity such as the restrictive permeability and control of leukocyte transmigration into brain parenchyma [10, 11]. Growing evidence demonstrates that inflammatory challenge results in an increase in the brain microvascular permeability [12]. The administration of cytokines, such as interleukin (IL)-1, tumor necrosis factor-α (TNFα), and IL-6, increases endothelial permeability [13, 14]. Astrocytes also play a critical role in maintaining the homeostasis of the BBB with respect to neuroglial cells. Astrocyte end-feet contain several channel proteins, such as the inwardly rectifying potassium (Kir) channel subunit Kir4.1, one of the integral parts of the orthogonal arrays of particles, which are required for the provision of optimal BBB properties [11]. Kir 4.1 channels, which are specifically expressed in astrocytes, play a key role in controlling spatial K^+^ concentration and regulating extracellular glutamate concentration at tripartite synapses [15, 16]. Astrocytes, via channels such as Kir4.1, can directly affect neural excitability and have been implicated in the pathogenesis of seizures and the development of epilepsy [15].

Necroptosis is a form of regulated necrosis, which differs from apoptosis and necrosis, and is mediated by death receptors such as TNFα receptor 1, receptor-interacting protein kinase (RIP)1, and RIP3, which activate the phosphorylation of mixed lineage kinase domain-like (MLKL) protein, thereby disturbing the integrity of the cells [17, 18]. The necroptosis pathway reportedly serves a crucial role in multiple pathologies that involve inflammatory processes such as sepsis, inflammatory bowel disease, and neurodegenerative diseases [19]. The necroptosis mechanism has attracted considerable attention from the research community owing to its importance in endothelial damage and BBB leakage after stroke [20, 21]. However, reports about necroptotic cell death signaling occurring in these BBB components following systemic inflammation have been few.

Sepsis and systemic inflammation induce the excessive production of proinflammatory factors and their rapidly corresponding neuroinflammation [7], which may result in brain vascular (e.g., BBB) injury and subsequent leakage [14]. Necroptosis is closely associated with and inflammation. Therefore, we hypothesized that necroptosis links inflammatory mediators responsible for systemic inflammation (e.g., TNFα) to changes in brain vascular barrier components (e.g., endothelial cells and astrocytes), and vascular integrity, thereby contributing to alteration in susceptibility to seizure. By employing an LPS-induced systemic inflammation mouse model, we found for role of TNFα-mediated necroptosis in cerebrovascular endothelial cell damage and astrocytic Kir4.1 dysregulation, which possibly were related to an increase in the susceptibility to seizures in mice following systemic inflammation.

## Materials and methods

### Animals and the lipopolysaccharide (LPS)-induced systemic inflammation model

Eight- to nine-week-old male C57BL/6 J mice were purchased from the National Lab Animal Center (Taiwan). The animals were housed in a pathogen-free room at 21 °C under a 12-h light/12-h dark artificial lighting cycle, with free access to feed. Age- and weight-matched animals were used for the experiments conducted in this study. All procedures were approved by the Animal Care and Use Committee of Changhua Christian Hospital (No. CCH-AE-100-012). To create a mouse model of systemic inflammation, mice were intraperitoneally injected with 4 mg/kg LPS (*Escherichia coli*, strain O111:B4, Calbiochem, San Diego, CA, USA), as previously described [7]. In the LPS-induced systemic inflammation experiments, a score for the severity (0 to 5) of sepsis sickness by general activity and response to stimuli [22] was used. Upon reaching a sickness score > 4 once or a score of 4 twice within 2 h, mice were immediately euthanized.

### Determination of susceptibility to seizure using kainic acid

To assess their susceptibility to seizure, mice were intraperitoneally injected with 3 or 20 mg/kg kainic acid (KA) 72 h after treatment with LPS (Sigma, St. Louis, MO, USA) or vehicle (normal saline). KA is a common proconvulsant agent used for the induction of seizures. Seizure activity was recorded on video during an observation period of 2 h after KA injection. Behavioral seizures were scored as per a previously defined scale [23] as follows: stage 0, no response; stage 1, lowering of the body position and hypoactivity; stage 2, automatic shaking of the body, whisker twitching, and sudden muscle or tail contraction; stage 3, repetitive scratching, head bobbing, or circling; stage 4, forelimb clonus and rearing and falling; stage 5, repetitive forelimb clonus and rearing and falling; and stage 6, severe generalized tonic–clonic seizure. In the behavior test, we recorded the severity score as per the aforementioned criterion for every 5 min over a 2-h *n* period. During a 2-h observation period following kainic acid treatment at 72 h after LPS injection, animals spontaneously died due to severe seizure or were killed at the planned experiment endpoint, that is, at 74 h after LPS injection.

### Plasma levels of tumor necrosis factor-α (TNFα) after peritoneal administration of LPS

To assess the acute effects of LPS on systemic inflammation with pretreatment with/without C87 (a TNFα receptor inhibitor) and GSK872 (a RIP3 kinase inhibitor), blood samples were obtained from the cheek of the mice 1 h after the i.p. injection of 4 mg/kg LPS. The plasma samples were stored at − 80 °C until they were assayed for TNFα concentrations using Duo set kits (R&D Systems, Minneapolis, MN, USA), based on the manufacturer’s instructions.

### Western blot analysis

For protein lysate extraction, the dissected hippocampus was homogenized and lysed in an ice-cold modified radioimmunoprecipitation assay buffer containing 150 mM NaCl; 50 mM Tris–HCl (pH 7.4); 1 mM phenylmethylsulfonyl fluoride; 1% Nonidet P-40; 1 mM EDTA; 10 μg/mL each of leupeptin, aprotinin, and pepstatin; 1 mM NaF; and 1 mM Na_3_VO_4_. Immunoblotting analysis was performed as previously described [7]. Briefly, 20–30 µg of total protein was loaded per lane onto 10–15% polyacrylamide gels. The gels were transferred to polyvinylidene difluoride membranes, which were probed with antibodies against proteins including c-Jun N-terminal kinases (JNK) (1:1000; #9258, Cell Signaling, Danvers, MA, USA), Bax (1:1000; #2772, Cell Signaling), cleaved Caspase 3 (1:500; ab13847; Abcam Cambridge, UK;), RIP3 (1:1000; #95702, Cell Signaling), RIP3 (phospho S227) (1: 1000; ab209384, Abcam), MLKL (1:500; ab196436, Abcam), MLKL (Ser125) (1:500; PA5-105677, Invitrogen, CA, USA), TNFα (1:1000; #11948, Cell Signaling), Na–K–Cl cotransporter 1 (NKCC1) (1:1000; #14581, Cell Signaling), KIR4.1 (1:500; sc-23637, Santa Cruz, CA, USA), and alpha-tubulin (1:1000; GTX628802, GeneTex, CA, USA), and beta-actin (1:2500; sc-47778, Santa Cruz) overnight at 4 °C, followed by pairing different secondary antibodies with horse radish peroxidase-conjugated goat anti-mouse and goat anti-rabbit at dilution of 1:10,000. Signals were visualized and quantified using the GeneGnome chemiluminescence imaging system (Syngene, Bengaluru, India).

### Immunofluorescence staining

Mice brains were harvested and soaked in 4% paraformaldehyde overnight and then dehydrated in 30% sucrose at 4 °C for 48 h. Serial 20-μm cryosections were cut, washed three times with phosphate-buffered saline (PBS), and then treated with sodium citrate buffer (10 mM sodium citrate, 0.05% tween 20, pH 6.0) at 80 °C for 30 min. The cryosections were washed with PBS three times after cool down and permeabilized with 0.15% Triton X-100 (Thermo Fisher Scientific, MA, USA) in commercial blocking buffer at room temperature (23–25 °C) for 30 min. The cryosections were hybridized with each primary antibody, including those against, Iba1 (1:200; GTX100042, GeneTex), GFAP (1:500; GTX85454, GeneTex), MLKL (Ser125) (1:100; PA5-105677, Invitrogen;), Kir4.1 (1:250; sc-23637; Santa Cruz), CD31 (1:800; ab24590, Abcam), and CD68 (1:300; MAB1435, Merck Millipore, MA, USA), overnight at 4 °C and then with different rhodamine- or Alexa-488-conjugated secondary antibodies, as previously described [7]. Sections were counterstained with 6-diamidino-2-phenylindole to identify the cell nucleus. Microfluorescence images were taken using an Olympus microscope (Olympus DP80® Dual CCD Microscope, Tokyo, Japan). Immunofluorescence images were used for the analysis of the staining density in the CA3 subregions using ImageJ software (NIH, Bethesda, MD, USA) [24]. The number of activated microglia and reactive astrocytes were counted using a stereological approach from three sections of the CA3 subregion in each hippocampus (*n* = 3 mice per group), employing an Olympus DP80® Dual CCD Microscope, as previously described [25]. Briefly, images were captured from the CA3 subregion at − 2.18 to − 2.54 mm from the bregma. The coordinates for the CA3 were taken from the 2.0- to 3.0-mm medial-to-lateral regions and the 2.0- to 3.2-mm dorsal-to-ventral regions. Activated microglia could be distinguished from resting microglia by their amoeboid appearance and significant enlargement. Reactive astrocytes had a hypertrophic morphology distinct from that of resting astrocytes. A single experimenter, who was blinded to the animal’s treatment protocol followed for each animal, performed the quantification of cells of interest using images taken at 200 × magnification in selected rectangular regions.

### Brain interstitial fluid microdialysis

Eight- to nine-week-old male C57BL/6 J mice were used for brain microdialysis assays, using procedures modified from previous studies [26, 27]. Mice were anesthetized with isoflurane for stereotaxic surgery to place a guide cannula (Plastics One, Roanoke, VA, USA) into the right hippocampus (anterior–posterior, − 2.3 mm; medial–lateral, 2.0 mm; and dorsal–ventral, − 2.0 mm relative to the bregma). One day postoperatively, mice (*n* = 36) were treated with either vehicle (0.25% dimethyl sulfoxide (DMSO)) or 2 mg/kg GSK872, and treated with saline or 4 mg/kg LPS 1 h later. Eighteen of these mice had CMA/12 probes (CMA/12 Elite, lengths 2 mm, CMA, Stockholm, Sweden) inserted 2 h before GSK872 treatment and were placed in a Plexiglas dialysis chamber. Then, the CMA/12 probes were immediately perfused with Ringer’s solution (147 mM Na^+^, 2.2 mM Ca^+2^, 4 mM K^+^, pH 7.0) at a flow rate of 2 µL/min, set to collect 60 µL of dialysate every 30 min for 6 h. Four days postoperatively, 18 more mice that had been treated with GSK872 and LPS had probes inserted into the guide cannulas placed to collect the dialysates once every 30 min for 2 h in each mouse. The extracellular K^+^ concentrations of these dialysates were measured using a flame atomic absorption spectrometer (Z6100 Hitachi, Japan). Potassium levels were calculated from a standard curve prepared from the standard solutions (Merck, Darmstadt, Germany). Glutamate concentrations were measured using an enzymatic colorimetric method using a microdialysis analyzer (CMA/600, Carnegie Medicine, Stockholm, Sweden).

### Statistical analysis

The means of two groups were compared using Student’s *t*-tests. The means of more than two groups were compared using one-way ANOVA, followed by Bonferroni post hoc tests. Differences in KA-induced seizure severity among treated mouse groups and extracellular potassium and glutamate changes among treated GSK872 mouse groups were assessed using two-way repeated measures ANOVA, adjusted using Bonferroni post hoc tests. Statistical analysis was performed using the GraphPad Prism version 7 software (GraphPad Software, San Diego, CA, USA, www.graphpad.com). All values are presented as mean ± standard error of the mean (SEM). Differences were considered statistically significant at *p* < 0.05.

## Results

### LPS-induced systemic inflammation increased the susceptibility to KA-induced seizures in mice

**Fig. 1:**
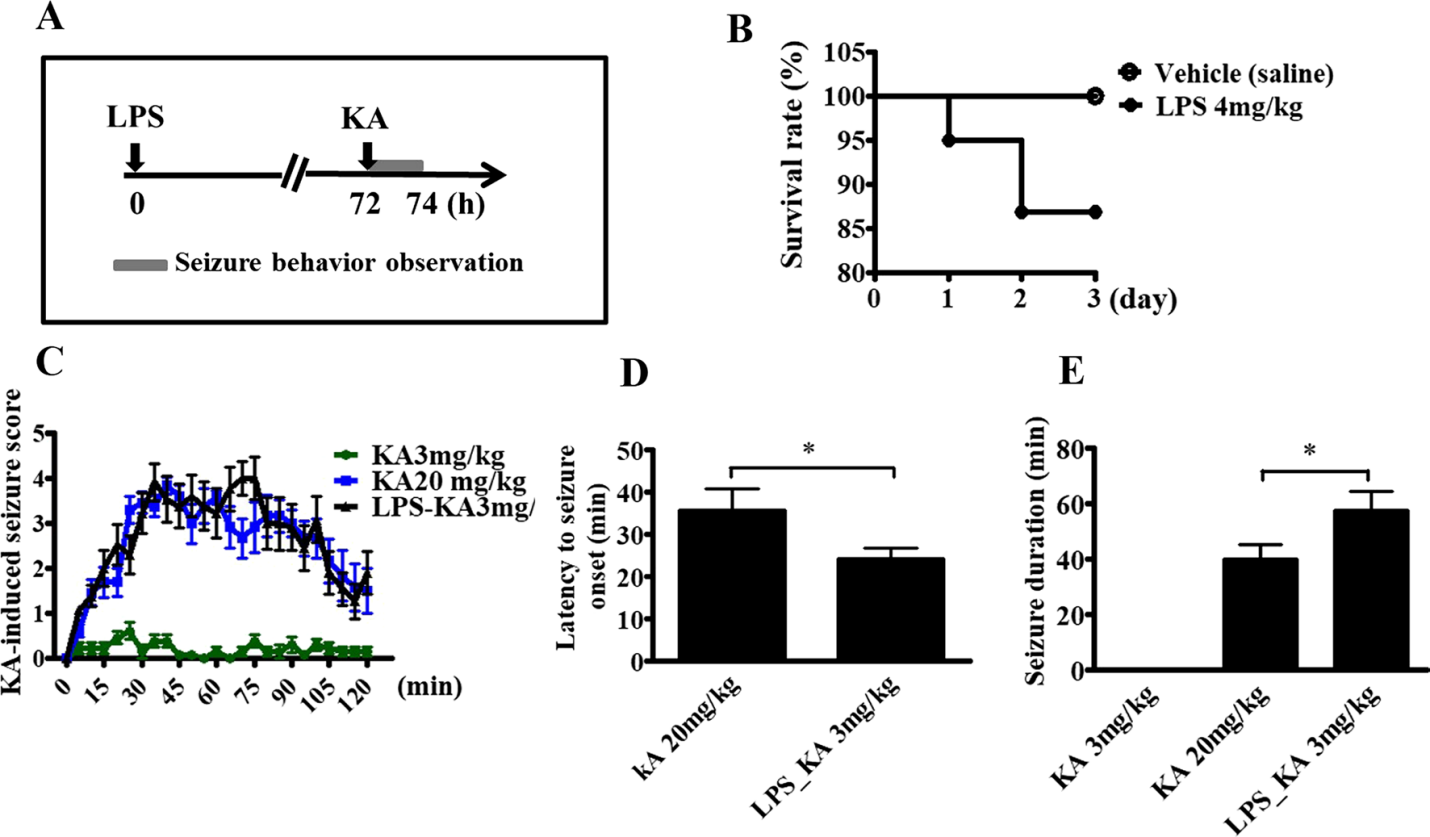
Increased susceptibility to kainic acid-induced seizures in mice following LPS-induced systemic inflammation. **A** The experimental protocol. Mice were injected intraperitoneally with vehicle (normal saline) or 4 mg/kg lipopolysaccharide (LPS). Three days later, seizure susceptibility to kainic acid (KA) administered intraperitoneally was evaluated. **B** The mortality rate of mice within 3 days after LPS or vehicle injection. **C** Seizure susceptibility of these mice was scored once every 5 min over the 2-h period following injection with 3 or 20 mg/kg KA (*n* = 13 mice per group). Using two-way repeated measures ANOVA analysis, the Bonferroni post hoc analysis revealed a significant difference between the 3-mg/kg-KA-saline-treated group and 20-mg/kg-KA-saline-treated group and between the 20-mg/kg-KA-saline-treated group and 3-mg/kg-KA- and LPS-treated groups. **D** The latency to initial seizure onset of stage 4 (i.e., tonic with or without clonic convulsion) or more after KA injection. Data are presented as mean ± SEM. Student’s *t*-test; *p < 0.05. **E** The total duration (min) of seizure behavior of stage 4 or more. Data are presented as mean ± SEM. Student’s *t*-test; 3-mg/kg-KA- and LPS-treated group vs. 20-mg/kg-KA- and saline-treated group; *p < 0.05

To explore the mechanisms underlying the onset and development of seizures following systemic inflammation, the mice model of systemic inflammation was used [7]. Seizure susceptibility was subsequently determined by scoring the severity and duration of the KA-induced seizure every 5 min for a 2-h period (Fig. 1A). The mortality rate among the mice was approximately 12% within 3 days after LPS injection (Fig. 1B). During a 2-h period after KA injection, one of 13 saline-treated mice administered 20 mg/kg KA and 2 out of 13 LPS-treated mice administered 3 mg/kg KA died from severe seizures. Among the groups treated with saline and either 3 or 20 mg/kg KA and the group treated with LPS and 3 mg/kg KA, two-way repeated measures ANOVA revealed that the main effect for these three groups yielded an *F* ratio value of *F*(2, 900) = 395.89, *p* < 0.0001 (Fig. 1C), indicating a significant difference in the susceptibility to KA-induced seizure between these three groups. Bonferroni post hoc tests further revealed a significant difference between the 3-mg/kg-KA–saline-treated group and 20-mg/kg–KA–saline-treated group (*F*(1, 600) = 860.18; *p* < 0.0001), but no difference between the 20-mg/kg-KA-saline-treated group and 3-mg/kg-KA- and LPS-treated groups (*F*(1, 600) = 1.17, *p* = 0.285). These results indicated that the LPS-treated mice showed increased susceptibility to 3-mg/kg-KA, which was similar to findings observed in saline- and 20-mg/kg-KA-treated mice (Fig. 1C). The latency to initial seizure onset that was defined as seizure score stage 4 (i.e., tonic with or without clonic convulsion) or more after KA administration was significantly decreased in LPS-treated mice that were administered with 3 mg/kg KA, compared with that in saline-treated mice that were administered with 20 mg/kg KA (Fig. 1D). All saline-treated mice that were administered with 3 mg/kg did not show tonic with or without clonic convulsion. We measured the duration of stage 4–6 seizures in the mice receiving LPS or vehicle control with KA. The seizure duration was 40.00 ± 5.31 min (mean ± SEM) for the saline- and 20-mg/kg-KA-treated groups and 57.69 ± 6.69 min for the LPS- and 3-mg/kg-KA-treated groups (Fig. 1E). Compared with the 20-mg/kg-KA-treated mice without LPS injection, the LPS-treated group had increased susceptibility to 3 mg/kg KA-induced seizures in the latency of initial seizure onset and seizure-behavior duration.

### LPS-induced systemic inflammation induced the programmed necroptosis and Kir4.1 dysregulation in the hippocampus

**Fig. 2:**
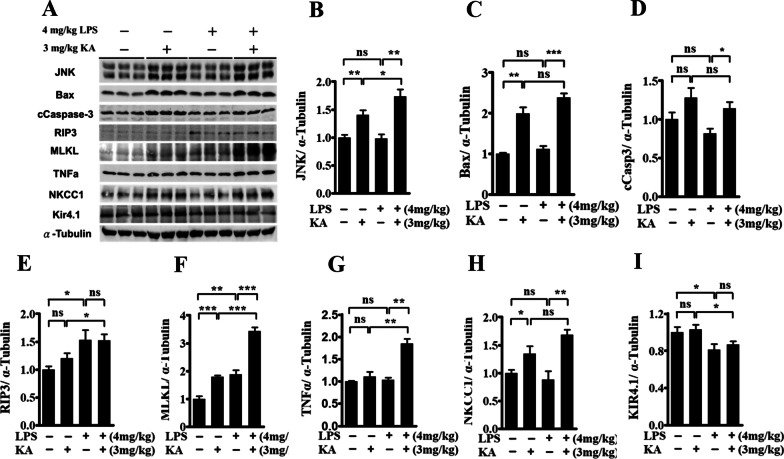
LPS-induced systemic inflammation induced the programmed necroptosis pathway and Kir4.1 dysregulation in the hippocampus. Three days after LPS or vehicle (saline) injection, 3 mg/kg kainic acid or vehicle (saline) was intraperitoneally administrated to the mice. Two hours later, the hippocampus was obtained for assessing the protein expression (*n* = 3 per group) using Western blots. **A** A representative Western blot images showing the specific bands for these proteins. An equal amount of protein sample (30 μg) obtained from the hippocampus homogenate was applied to each lane, and α-tubulin protein was used as an internal control. **B**–**D** Bar graph showing the densitometric analysis of the molecules JNK, Bax, and cCaspase 3 involved in the apoptotic pathway; **E**–**G** of the proteins phosphated RIP3, phosphated MLKL, and TNFα in the necroptosis pathway; and (**H** and **I**) of the proteins NCKK1 and Kir4.1 in ion channels, normalized to α-tubulin expression. Each bar represents the mean ± SEM. One-way ANOVA; Bonferroni post hoc test among the groups; *p < 0.05, **p < 0.01, and ***p < 0.001

We determined the role of several signaling pathways that could be involved in the mechanisms of neuronal hyperexcitability following systemic inflammation, including pathways involved in apoptosis, necroptosis, and ion channel changes [28, 29]. Seventy-two hours after injection with vehicle (saline) or 4 mg/kg LPS, the mice were intraperitoneally administered either the vehicle (saline) or 3 mg/kg KA. Two hours later, the hippocampus was used for the assessment of the expression of proteins involved in the apoptosis and necroptosis pathways (*n* = 3 mice per group) and of astrocytic ion channel protein (*n* = 3 mice per group) using Western blotting. Among these four groups of saline- or LPS-treated mice that were administered with the vehicle or KA, the protein levels of JNK, Bax, and cCaspase 3, which are part of the apoptosis pathway, were significantly increased following KA treatment, but no difference was noted in the mice treated with LPS alone (Fig. 2A–D). The protein levels of phosphorylated RIP3 and phosphorylated MLKL, from the necroptosis pathway, were increased in LPS-treated mice, enhanced by KA treatment, compared with those in the vehicle-treated mice (Fig. 2A, E, F). The levels of TNFα were markedly enhanced after treatment with 3 mg/kg KA only in the LPS-treared mice (Fig. 2A, G). The protein levels of Kir4.1, but not NKCC1, were significantly decreased in the LPS-treated mice, compared with those in vehicle-treated mice (Fig. 2H, I).

### Treatment with C87 and GSK872 attenuated the increased susceptibility to kainic acid (KA)-induced seizures in mice following LPS injection

**Fig. 3:**
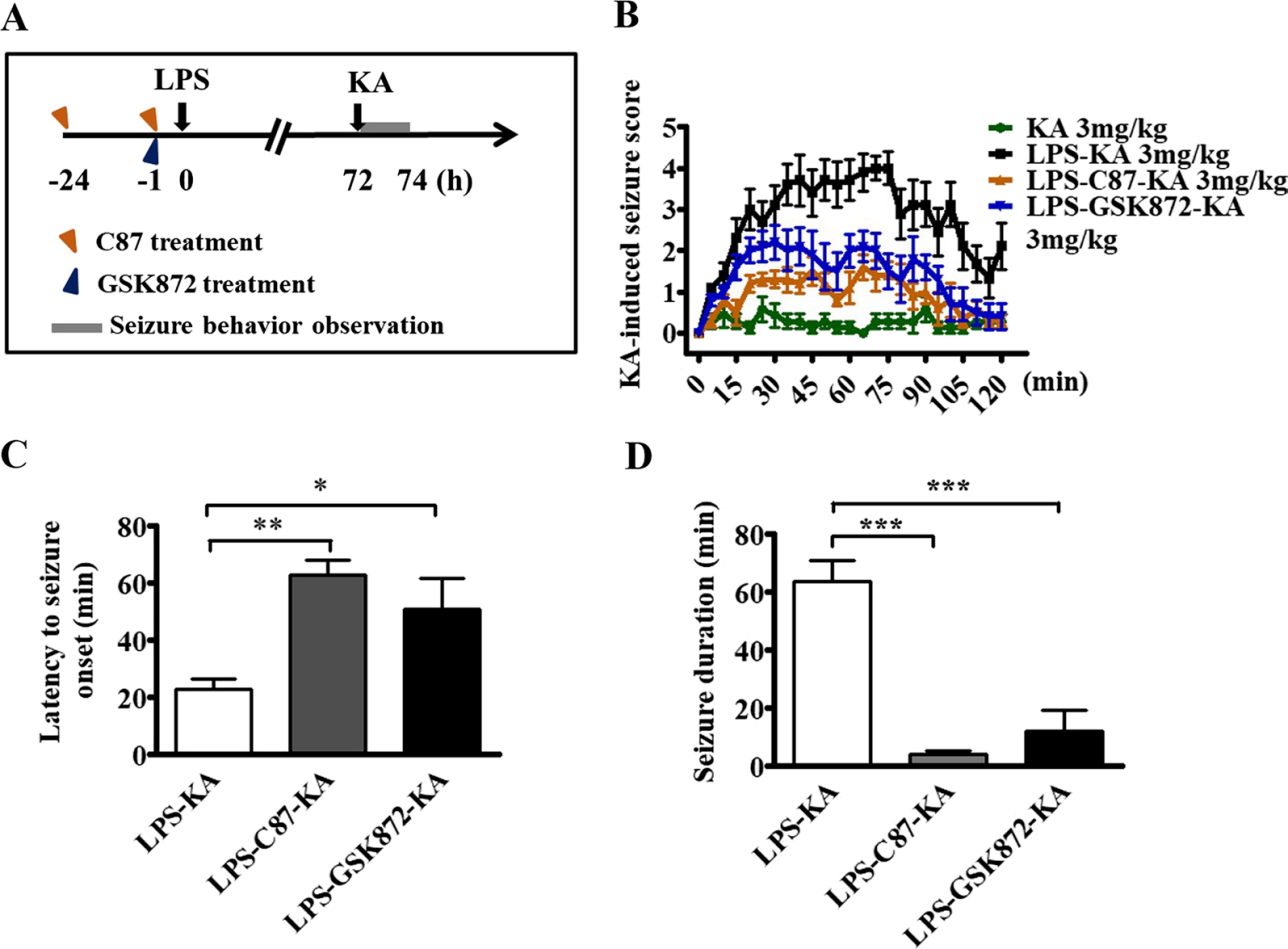
C87 and GSK872 pretreatment attenuated the increased susceptibility to kainic acid-induced seizures in mice following LPS injection. **A** The experimental protocol. Mice were injected intraperitoneally with 12.5 mg/kg C87, a TNFα receptor inhibitor, given at 24 and 1 h before 4 mg/kg LPS i.p. injection or with 2 mg/kg GSK872, a RIP3 inhibitor, at 1 h before LPS injection. Three days later, seizure susceptibility to 3 mg/kg kainic acid (KA) was evaluated (*n* = 7–10 mice per group). **B** Seizure susceptibility of treated mice scored once every 5 min over the 2-h period following KA injection. Using two-way repeated measures ANOVA analysis, the Bonferroni post hoc analysis revealed a significant difference between C87-treated mice and GSK872-treated mice, compared with vehicle-treated mice following LPS injection, and a significant difference between C87- and GSK872-treated mice. **C** Latency to initial seizure onset (tonic with or without clonic convulsion) after KA administration. Data are presented as mean ± SEM. One-way ANOVA; Bonferroni post hoc test vs. vehicle- and LPS-treated groups; *p < 0.05, **p < 0.01. **D** The total duration (min) of seizure behavior of stage 4 or more. Data are presented as mean ± SEM. One-way ANOVA; Bonferroni post hoc test vs. vehicle- and LPS-treated groups; ***p < 0.001

TNFα is rapidly released and is one of the most abundant mediators of inflammation in the peripheral blood after infection or exposure to LPS [7]. TNFα has been implicated in the pathogenesis of several inflammation-related diseases, such as vascular leaks, via different signaling pathways [30]. Based on the aforementioned evidence (Fig. 2), TNFα-dependent necroptosis appeared to be involved in the increase of LPS-induced susceptibility to seizure. Therefore, a TNFα receptor inhibitor, C87, and a RIP3 inhibitor, GSK872, were used to investigate the role of TNFα-dependent necroptosis in LPS-associated susceptibility to seizure. Mice were injected intraperitoneally with two doses of C87 (2 mg/kg, i.p.) at 1 and 24 h, or one dose of GSK872 (2 mg/kg, i.p.) at 1 h, before LPS injection (Fig. 3A). The mortality rate of the mice was approximately 12% within 3 days after the administration of LPS only, and no deaths were recorded in mice receiving either C87 or GSK872 with LPS treatment. Seventy-two hours after LPS injection, 3 mg/kg KA was administered to evaluate the susceptibility of these mice to seizure (*n* = 7–10 mice per group) by scoring the seizures once every 5 min for 2 h. During a 2-h period after KA treatment, one of the 10 LPS-treated mice that were administered with KA died because of severe seizures; no death was observed in the LPS-treated groups receiving C87 (*n* = 10) or GSK872 (*n* = 10), as well as in the saline-treated group (*n* = 7). Two-way repeated measures ANOVA revealed that the main effect for these four groups yielded an *F* ratio of *F*(3, 825) = 192.65, *p* < 0.0001 (Fig. 3B). The Bonferroni post-tests analysis further revealed that there was a significant difference between the C87-treated group (*F*(1, 450) = 273.49, *p* < 0.0001) and the GSK872-treated group (*F*(1, 450) = 117.60, *p* < 0.0001) compared with the vehicle-treated mice following LPS injection. These results indicated that either C87 treatment or GSK872 treatment attenuated the susceptibility of mice to KA-induced seizures following LPS injection. There was a significant difference between C87-treated mice and GSK872-treated mice (*F*(1, 450) = 25.56, *p* < 0.0001), indicating that C87 treatment was better than GSK872 treatment for decreasing the susceptibility to seizures in the LPS-treated mice. The latency to initial seizure onset (Fig. 3C) and seizure duration during a 2-h period (Fig. 3D) after administration of KA (stage 4 or more) were considerably attenuated by either C87 or GSK872 treatment in LPS-treated mice, compared with those in vehicle-treated mice. Half of the mice treated with C87 and GSK872 were not observed to have tonic with or without clonic seizure (i.e., stage 4).

### Treatment with C87 and GSK872 attenuated monocyte infiltration, endothelial necroptosis, and astrocytic Kir4.1 downregulation in the hippocampus of mice treated with LPS

**Fig. 4:**
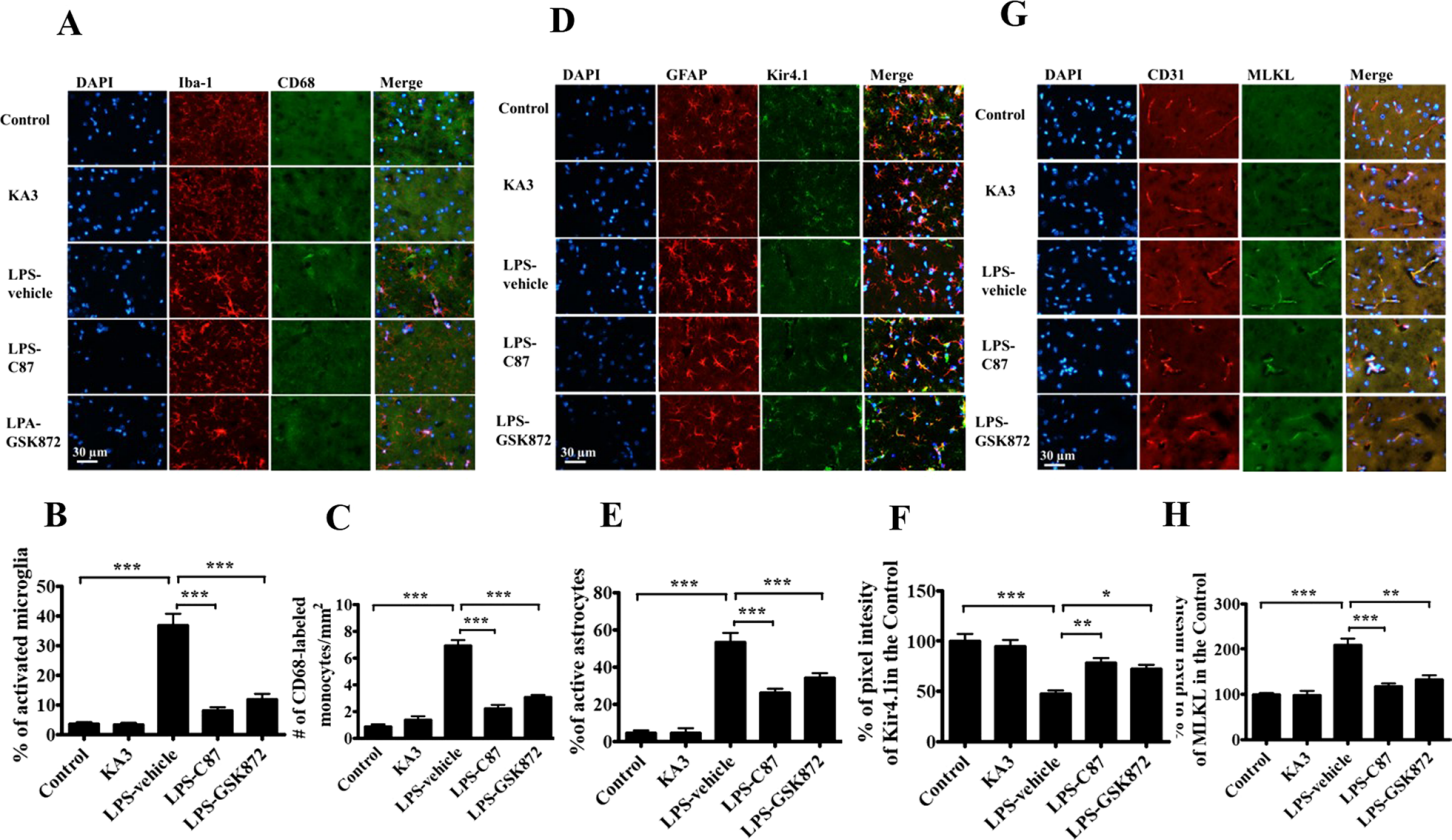
C87 and GSK872 pretreatment alleviated glia activation, monocyte infiltration, endothelial cell MLKL activity and downregulated KIR4.1 expression. Mice were treated with two doses of C87, a TNFα receptor inhibitor, or vehicle at 24 and 1 h before 4 mg/kg LPS injection (i.p.), or with GSK872, a RIP3 inhibitor, at 1 h before LPS was administrated, and then killed 72 h after LPS administration. Hippocampal tissues of these mice were prepared for immunostaining (*n* = 3 per group). **A** Representative images of microglia stained with Iba-1 and monocytes stained with CD68 in the CA3 region of the hippocampus. Increased cell size and irregular shape were identified as the activated microglia. **B** The proportions of activated microglia and **C** the number/mm^2^ of CD68-positive cells in the CA3 of the hippocampus were estimated. **D** Representative images of astrocytes stained with GFAP and Kir4.1. Hypertrophic morphology identified as the activated astrocytes. **E** The proportions of activated GFAP-positive astrocytes and **F** the densitometric analysis of Kir4.1- and GFAP-positive protein levels in CA3 hippocampus. **G** Representative images of brain endothelial cells stained with CD31 and p-MLKL in the CA3 regions and **H** densitometric analysis of co-localized CD31- and p-MLKL-positive protein levels. Densitometry was semi-quantified using ImageJ software. Data represent the mean ± SEM of values from three animals per treatment group. One-way ANOVA; Bonferroni post hoc test vs. vehicle-treated mouse group; *p < 0.05, **p < 0.01, ***p < 0.001

To investigate the effect of the TNFα-dependent necroptosis signal pathway on the integrity of the cerebral vasculature following LPS injection, we examined the effects of C87 and GSK872 treatment on the cerebral vascular integrity and neuroinflammation. Mice were dosed with C87 at 24 and 1 h before LPS injection, or one dose of GSK872 at 1 h before LPS, or vehicle and were then killed at 72 h after LPS treatment. Hippocampus sections were prepared for immunostaining with Iba-1 antibody for microglia, CD68 antibody for monocytes, GFAP antibody for astrocytes, and Kir4.1 antibody for channel proteins. Activated microglia were identified by their increased cell size and irregular shape. The percentage of activated microglia (Fig. 4A, B) and infiltrated monocytes (Fig. 4C) in the CA3 regions of the hippocampus was significantly increased in the LPS-treated group, which was attenuated by pretreatment with either C87 or GSK872. Reactive astrocytes indicated the presence of hypertrophic morphology. The percentage of active astrocytes was significantly higher in the LPS-treated mice than in the C87- or GSK-872-treated mice (Fig. 4D, E). The proportion of GFAP-positive cells co-localized with Kir4.1 was constant in the control and KA-treated mice but markedly decreased in the LPS-treated mice (Fig. 4F). For determining the MLKL activity of cerebral vascular endothelial cells, immunostaining was performed using CD31 antibody for the endothelial cells and p-MLKL antibody for the bioactivity (Fig. 4G). The proportion of CD31-positive cells co-localized with p-MLKL was lower in control mice, but markedly increased in LPS-treated mice (Fig. 4H). C87 and GSK872 pretreatment of LPS-treated mice reversed the phenomenon of enhanced p-MLKL-positive staining in cerebral vascular endothelial cells and decreased Kir4.1-positive staining in astrocytes.

### GSK872 suppressed RIP3-mediated necroptosis and restored Kir4.1 protein expression in mice within 3 days after LPS injection

**Fig. 5:**
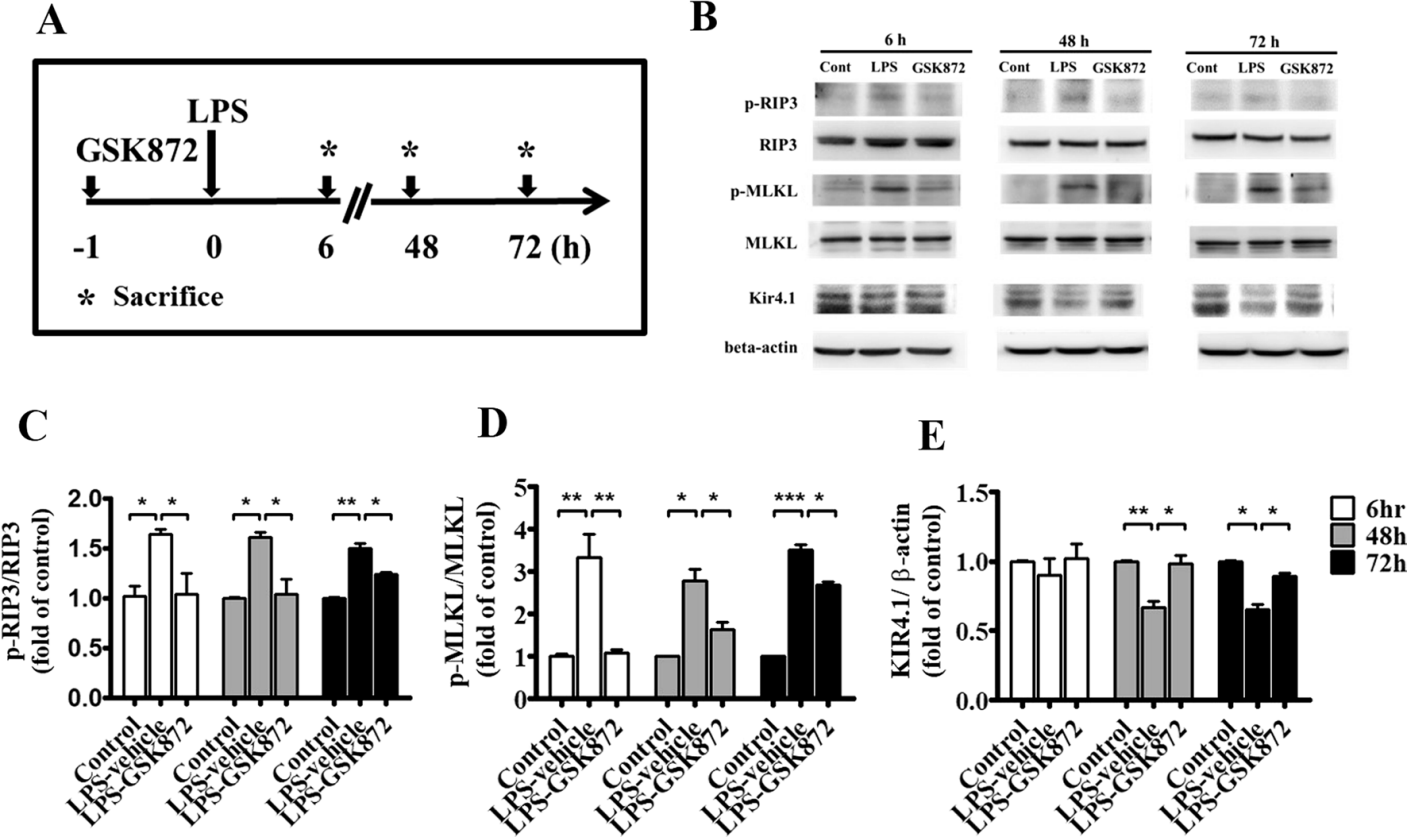
GSK872 suppressed RIP3-mediated necroptosis and restored Kir4.1 protein expression in mice within 3 days after LPS injection. **A** The experimental protocol. Mice were treated with GSK872 (2 mg/kg, i.p.) or vehicle (0.25% DMSO) 1 h before 4 mg/kg LPS was given. The hippocampus was obtained to assess the protein levels at 6, 48, and 72 h after LPS injection (*n* = 3 per group, each time point). The expression levels of p-RIP3, RIP3, p-MLKL, and MLKL detected RIP3-mediated necroptosis. **B** A representative Western blot images showing the specific bands for these proteins and Kir4.1 protein. An equal amount of protein sample (20 μg) obtained from the hippocampus homogenate was applied to each lane, and β-actin protein was used as the internal control. **C**–**E** Bar graphs showing the densitometric analysis of the molecules p-RIP3 and total RIP3 (**C**), p-MLKL and total MLKL (**D**), and Kir4.1 (**E**), normalized to total RIP3, total MLKL or β-actin expression, respectively. Each bar represents the mean ± SEM. One-way ANOVA; Bonferroni post hoc test vs. saline- and vehicle-treated groups or LPS- and vehicle-treated groups; *p < 0.05, **p < 0.01, and ***p < 0.001

We examined the effects of GSK872 on dynamic changes in RIP3-mediated necroptosis and Kir4.1 protein within 3 days after induction by LPS. Mice were pretreated with GSK872 (2 mg/kg, i.p.) or vehicle (0.25% DMSO) 1 h before the administration of LPS (Fig. 5A). The hippocampus was obtained used for the assessment of the expression of RIP3, MLKL and Kir4.1 proteins at 6, 48, and 72 h after LPS injection (*n* = 3 per group for each time point). The protein levels of p-RIP3 and p-MLKL were significantly higher (Fig. 5B–D) and those of Kir4.1 were significantly lower than those observed in the hippocampus of mice administered with the vehicle (Fig. 5B, E) at these time points within 3 days after LPS treatment. GSK872 treatment improved the RIP3-mediated necroptosis and the parallel decrease in the expression of Kir4.1 ion channel proteins.

### GSK872 attenuated the increased extracellular concentrations of potassium and glutamate in the hippocampus within 3 days after LPS injection

**Fig. 6:**
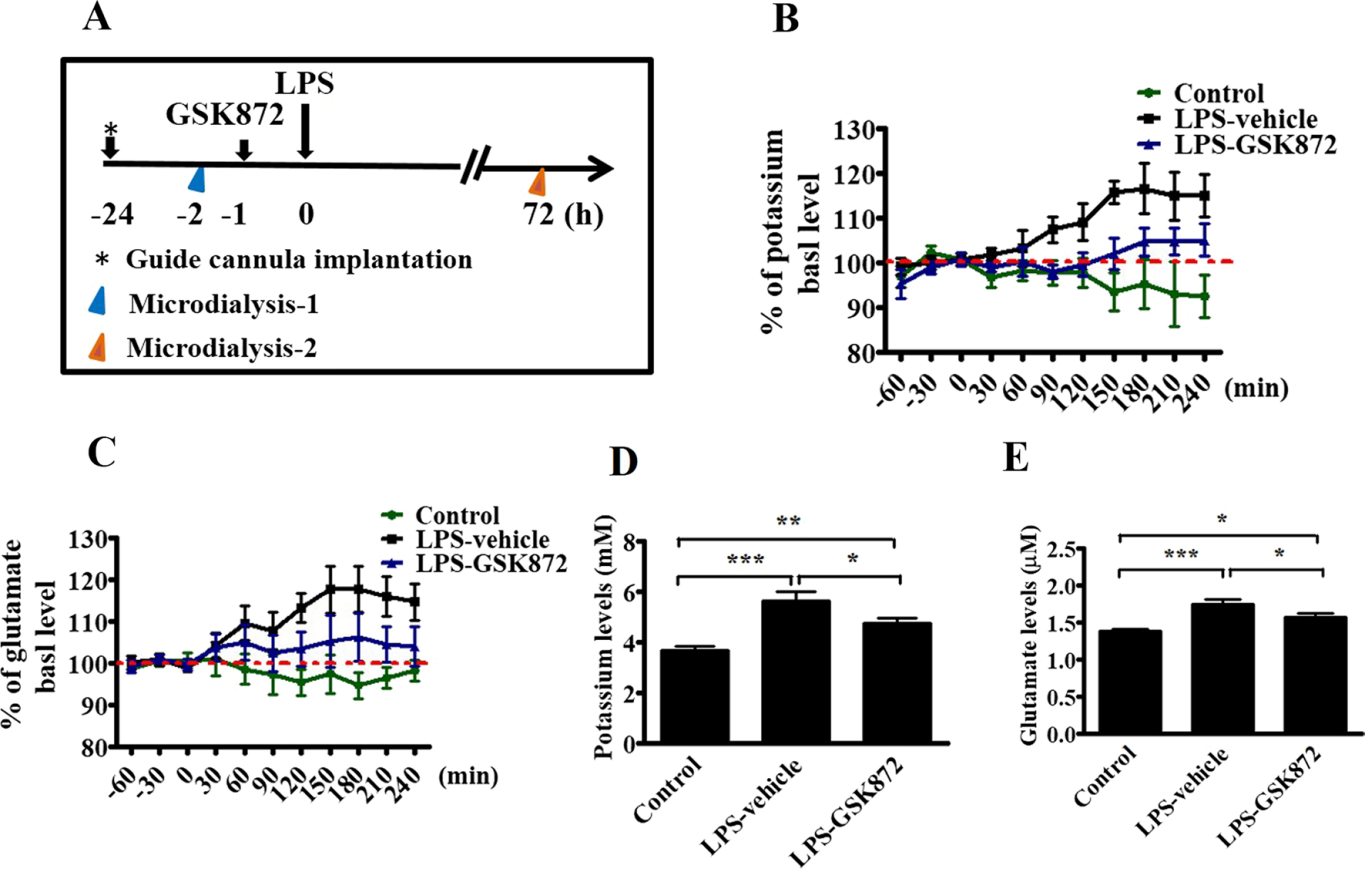
GSK872 attenuated the increased extracellular levels of potassium and glutamate in the hippocampus within 72 h after LPS injection. **A** The experimental protocol. One day after the chronic indwelling guide cannula was implanted in the hippocampus, mice were treated with GSK872 (2 mg/kg, i.p.) or vehicle (0.25% DMSO) 1 h before 4 mg/kg LPS was given. Microdialysis experiments were performed 1 h before GSK872 injection. Samples were collected once every 30 min for 5 h from each mouse after 1 h stabilization, and the first three samples were defined as the basal levels (100%). **B** and **C** The time course concentrations of extracellular potassium and glutamate in the hippocampus after GSK872 plus LPS treatment (*n* = 6 per group). Data represent percentages (mean ± standard error of the mean (SEM)) of values. Using two-way repeated measures ANOVA analysis, Bonferroni post hoc analysis revealed that GSK872 significantly inhibited the increase in potassium levels and glutamate levels induced by LPS given systemically. At 72 h after LPS injection, additional microdialysis experiments were performed in treated mice with sample collection once every 30 min for 2 h from each mouse after 1 h stabilization. **D** and **E** Mean levels of extracellular potassium and glutamate in the hippocampus 72 h after LPS treatment (*n* = 6 per group). Data represent the mean ± SEM of values. One-way ANOVA; Bonferroni post hoc test; *p < 0.05, **p < 0.01, ***p < 0.001

Microdialysis was used for continuous measurement of free and unbound analyte levels in the extracellular fluid in mice treated with GSK872 and LPS. One day after the cannula was implanted in the hippocampus, the mice were treated with GSK872 (2 mg/kg, i.p.) or vehicle (0.25% DMSO, i.p.) (*n* = 6 per group) 1 h before the administration of either saline or 4 mg/kg LPS (Fig. 6A). The mean of the first three samples immediately before LPS administration was defined as the basal levels (100%) of extracellular potassium and glutamate in the hippocampus. Within 5 h after GSK872 and LPS injections, the levels of extracellular potassium and glutamate increased (Fig. 6B, C). Two-way repeated measures ANOVA revealed that the main effect for these three groups yielded an *F* ratio of *F*(2, 165) = 28.66, *p* < 0.0001 on potassium levels (Fig. 5B) and *F*(2, 165) = 24.31, *p* < 0.0001 on glutamate levels (Fig. 6C). The Bonferroni post-test analysis further revealed a significant difference in potassium (*F*(1, 110) = 25.87, *p* < 0.0001) and glutamate levels (*F*(1, 110) = 12.59, *p* = 0.0006) in the GSK872-treated group compared with that in the vehicle-treated group following LPS injection. These findings indicated that GSK872 treatment attenuated the changes in the levels of these extracellular molecules induced by LPS. Microdialysis experiments were also performed in the treated mice (*n* = 6 per group) 72 h after LPS injection, with samples collected once every 30 min for 2 h from each mouse after 1 h stabilization. The mean levels of extracellular potassium (Fig. 6D) and glutamate (Fig. 6E) were significantly higher in the LPS-treated group than in the vehicle-treated group and GSK872-treated group. These results indicated that GSK872 treatment attenuated LPS-induced aggravated potassium and glutamate changes within 3 days after LPS injection.

## Discussion

In this study, sepsis and systemic inflammation showed a consistent association with increased susceptibility to seizure and corresponding neuro-inflammation such as the activation of microglia and astroglia in the brain [3–5, 7]. We also demonstrated that the TNFα-dependent necroptosis signaling pathway governed the changes in the cerebrovascular endothelial cell damage and astrocytic ion channel Kir4.1 dysregulation associated with subsequent neuronal hyperexcitability and the induction of seizures.

Our previous study showed increased susceptibility in mice to seizures induced by utilizing the proconvulsant pentylenetetrazole following the administration of LPS [7]. The present study further showed that the susceptibility to low-dose (3 mg/kg, i.p.) KA-induced seizure in mice with LPS injection was similar but more severe in initial latency of seizure onset and tonic–clonic seizure duration than those in mice injected with a high dose (20 mg/kg, i.p.) of KA with saline injection (Fig. 1). These findings indicate that systemic inflammation increased neuronal excitability, which in turn reduced the threshold at which seizures are initiated by proconvulsants, or induced the onset of seizures. Apart from sepsis-induced neuroinflammation and the subsequent production in the brain of proconvulsive cytokines in the brain, such as TNFα and IL-1β, which may be involved in neuronal activity changes [7], the results of this study suggest that the dysregulation of the ion channel Kir4.1 of astrocytes plays an important role in the underlying mechanism for changes in seizure threshold in LPS-treated mice. The inwardly rectifying K^+^ channels, Kir4.1, are enriched on the processes of astrocytes surrounding the synapses and blood vessels in the brain [31]. In neuronal excitation, the astrocytic Kir4.1 channels play a major role in extracellular potassium (K^+^) buffering to maintain the homeostasis of the neuronal microenvironment [32]. Diminished Kir4.1 buffering capabilities, such as pharmacological or genetic inhibition and downregulation, may induce membrane hypo-polarization coupled to reduced glutamate clearance in astrocytes, leading to neuronal hyperexcitability [32, 33]. There is increasing evidence that strongly suggests that astrocytic Kir4.1 channels are involved in the development of seizure and epilepsy [15, 34, 35]. The present study found for the first time that downregulation of Kir4.1 in astrocytes combined with the increase in extracellular potassium and glutamate levels may be involved in the LPS-induced decreased seizure threshold in mice. Both in vitro and in vivo studies suggest that primary mediators of the inflammatory response, such IL-1β, influence the downregulation of Kir4.1 transcription and protein expression in astrocytes [36, 37]. The present study further demonstrated that Kir4.1 dysregulation in astrocytes induced by systemic inflammation could be restored by the inhibition of the TNFα-mediated necroptosis signaling pathway on brain endothelial cells. Interestingly, a significant increase of the extracellular levels of potassium and glutamate levels within 4 h after LPS injection seems not to be correlated with the changes in the downregulated Kir4.1 protein levels at 6 h after administration of LPS (Fig. 6). Since extracellular potassium regulation are highly dependent on the function of Kir4.1 channels on astrocytes in brain [16], this phenomenon suggests the possibility that a decrease in the biological activity of Kir4.1 channels in hippocampus may happen earlier than the significant downregulation of Kir4.1 protein after systemic inflammation.

The BBB aids in the regulation of the reciprocal peripheral blood-to-brain exchange of molecules and immune cells to maintain a tightly stable microenvironment for the CNS. Dysfunction of the BBB disrupts hemostasis, resulting in the pathological development of seizure disorders and other neurological disorders [38, 39]. During systemic inflammation, the components of the BBB could get altered at histological and/or molecular levels [14]. Endothelial cells lining the inner surface of blood vessels are a key component of the BBB, and damage to these cells during systemic inflammation may contribute to barrier dysfunction, whereas the other BBB components, including astrocytes, pericytes, and microglia/macrophages, do not appear to contribute much to the LPS-mediated disruption of the BBB [40]. In the present study, pretreatment with the TNFα receptor inhibitor C87 abolished endothelial necroptosis with a parallel change in corresponding neuroinflammation and Kir4.1 dysregulation. By excluding the possibility that C87 treatment alleviates LPS-induced circulating TNF-α levels (see Additional file 1: Fig. S1), these results strongly indicate that proinflammatory TNFα is a key factor involved in cerebral endothelial cell damage and changes in the vascular permeability of the brain (see Additional file 2: Fig. S2) in the event of systemic inflammation.

Upon binding to TNFα receptor 1, TNFα triggers a range of signaling pathways for the regulation of bioactivity in various cell types and tissues. TNFα-mediated activation may therefore affect the brain endothelial cells and BBB components [30]. Both in vitro and in vivo studies show that TNFα may induce endothelial cell injury and death via the apoptosis and necroptosis pathways [41, 42] and disrupt endothelial tight junction barriers via the targeting of different pathways, such as the Nuclear factor-kappa B pathway [43, 44], thereby increasing endothelial leakage. The present study found that both the inhibition of TNFα receptor by C87 and RIP3 necroptosis by GSK872 considerably attenuated the increased susceptibility to seizures in LPS-treated mice (Fig. 3), although substantial differences were noted between these two treatments. This phenomenon can be explained by the possibility that TNFα targets the components of the BBB more broadly. RIP3 inhibition might also affect apoptosis in some situations owing to the possibility of cross-talk between necroptosis and apoptosis [45]. Given that our data showed that LPS did not increase the level of cCasp3 in mouse brains 72 h after injection (Fig. 2A, D), the contribution of LPS-triggered apoptotic death of brain endothelial cells following necroptosis to the disruption of vascular integrity, if any, could be not significant. Therefore, among the signaling pathways involving TNFα, our data suggest that endothelial necroptosis plays a critical role in changes in seizure susceptibility in the context of TNFα-induced inflammatory events, including astrocytic ionic Kir4.1 channels and vascular barrier dysfunction.

TNFα production peaked in the peripheral blood at 1 h after LPS injection [7] and in the brain at approximately 30 h [46]. Evans Blue-measured BBB leakage was observed early, within 6 h after LPS injection (see Additional file 2: Fig. S2), which corresponded to the finding of increased RIP3-mediated necroptosis (Fig. 5) and extracellular potassium and glutamate levels within 4 h after LPS injection (Fig. 6). The results support the contention that systemic TNFα is an early key peripheral proinflammatory factor causing the disruption of brain vascular integrity during systemic inflammation, although it could not fully excluded the possibility that the TNFα derived from activated microglia [20]. In the CNS, TNFα consistently returned to the basal levels by 72 h after administration of LPS (Fig. 2G). KA treatment could not increase the brain levels of TNFα in saline-treated mice; however, the TNFα levels rapidly increased in the LPS-treated mice (Fig. 2G) [47, 48] and the MLKL activity was enhanced in necroptosis in mice 72 h after LPS injection (Fig. 2F). Although the detailed mechanisms of this phenomenon are not completely understood, severe seizures may induce brain injury via a process of necroptosis involving MLKL [49]. These results suggest that a seizure threshold low enough to easily induce severe seizure in LPS-treated mice and increased levels of TNFα after KA treatment contributes to additional MLKL-executed necroptosis in the brain (Fig. 2F).

Necroptosis is a type of programmed cell death with necrosis and is involved in a variety of biological processes, including inflammation and immune responses. Accumulating evidence suggests that the TNFα-mediated necroptotic pathway is a potential therapeutic target in the treatment of inflammatory diseases [50, 51]. Over the past years, several types of inhibitors targeting the kinase activity of necroptosis, such as RIP3, have been reported [51, 52]. In the present study, GSK872, an RIP3 inhibitor, reduced the phosphorylation of MLKL and the programmed necrosis of brain endothelial cells, supporting the suggestion that necroptosis is a therapeutic target with aim of preventing endothelial cell damage from systemic inflammation. RIP3 is indispensable in the TNFα-stimulated necroptosis pathway and can also promote non-necroptotic pathways such as inflammation activation and cytokine IL-1β production through the stimulation of Toll-like receptors [50]. Therefore, the effects of RIP3 inhibition on the improvement of seizure susceptibility associated with systemic inflammation could be partially due to non-necroptotic anti-inflammation, although MLKL activation in brain endothelial cells was considerably attenuated in our observations. Further determination of the function of RIP3 that drives the signaling pathways, such as those of inflammation and necroptosis, in each disease condition will be vital. In the future, studies exploiting more specific inhibitors of RIP3 and MLKL kinases may provide crucial insight into the prevention of necroptosis-associated brain endothelia damage and neuroinflammation following sepsis.

## Conclusions

Our results showed that TNFα-mediated necroptosis induced brain endothelial damage, neuroinflammation and astrocyte Kir4.1 dysregulation, which may coalesce to contribute to the increased seizure susceptibility in LPS-treated mice. Pharmacologic inhibition targeting elements of the necroptosis pathway, such as TNFα receptor and RIP3 kinases, reduced brain endothelial cell damage and improved seizure threshold in mice with systemic inflammation. This evidence may indicate a promising therapeutic approach to reduce sepsis-associated brain endothelial injury, astrocyte ion channel dysfunction, and subsequent neuronal excitability.

## Supplementary Information

**Additional file 1: Figure S1.** C87 and GSK872 pretreatment had no significant effects on plasma levels of TNFα after LPS injection. Mice were treated with vehicle, 2 doses of TNFα receptor inhibitor C87 (12.5/kg, i.p.) at 24 and 1 h before 4 mg/kg LPS injection (i.p.), or with a RIP3 inhibitor GSK872 (2 mg/kg, i.p.) at 1 h before LPS was administered, and then blood samples were obtained from the cheek of each mouse 1 h after LPS injection. The TNFα levels of the plasma samples were significantly high in the LPS-treated groups with/without C87 and GSK872, but there was no difference between mice pretreated with C87 or GSK872 and vehicle-treated mice following LPS injection. Data are presented as mean ± SEM; *n* = 7 per group. One-way ANOVA; Bonferroni post hoc test vs. vehicle- and LPS-treated mice; ***p < 0.001.

**Additional file 2: Figure S2.** Effects of GSK872 and C87 pretreatment on brain vascular permeability in LPS-treated mice (A) The experimental protocol. The mice were injected with 3% Evans blue dye (i.p.) 2 h after vehicle (saline) and LPS (4 mg/kg, i.p.) was administrated, and the brains were obtained 4 h later. (B) Representative photographs of brains and coronal brain sections with Evans blue extravasation. Evans Blue leakage into brain was measured by spectrophotometer at 620 nm and quantified according to a standard curve. The results were presented as µg of Evans Blue per mg of dried brain tissue. (C) The density levels of Evans blue leakage in hippocampus was higher in LPS-treated mice, which was significantly reduced in C87 and GSK872 pretreated mice (*n* = 3 per group). Data represent the mean ± SEM of values. One way ANOVA; ***p < 0.001; **p < 0.01.

## Data Availability

The datasets supporting the conclusions of this article are included within the article and its Additional files. All material used in this manuscript will be made available to researchers subject to confidentiality.

## Abbreviations

BBB: Blood–brain barrier
LPS: Lipopolysaccharide
TNFα: Tumor necrosis factor-α
i.p.: Intraperitoneal
CNS: Central nervous systems
IL: Interleukin
Kir: Inwardly rectifying potassium
RIP: Receptor-interacting protein kinase
MLKL: Mixed lineage kinase domain-like
KA: Kainic acid
