# Supplementary figures and images for: TNFα-mediated necroptosis in brain endothelial cells as a potential mechanism of increased seizure susceptibility in mice following systemic inflammation
Source: J Neuroinflammation. 2022 Feb 2;19:29. doi: 10.1186/s12974-022-02406-0 (PMC8809013; doi:10.1186/s12974-022-02406-0)

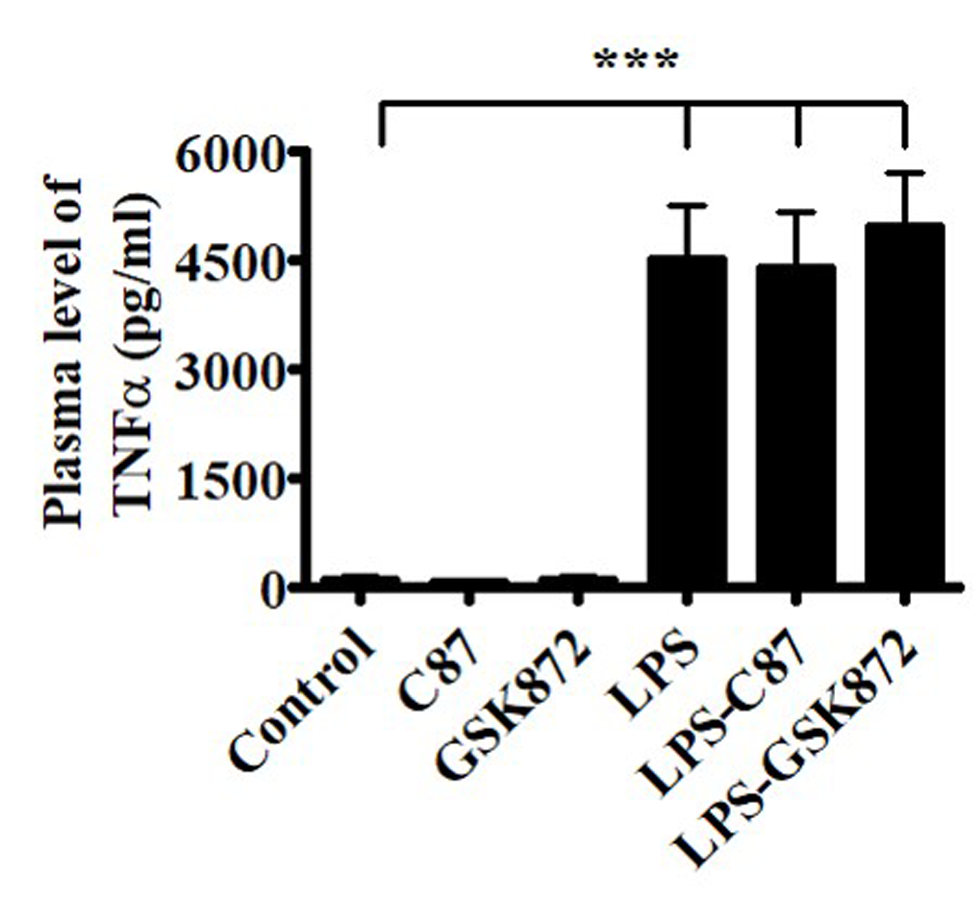

Supplement: Supplementary file 1 — Additional file 1: Figure S1. C87 and GSK872 pretreatment had no significant effects on plasma levels of TNFα after LPS injection. Mice were treated with vehicle, 2 doses of TNFα receptor inhibitor C87 (12.5/kg, i.p.) at 24 and 1 h before 4 mg/kg LPS injection (i.p.), or with a RIP3 inhibitor GSK872 (2 mg/kg, i.p.) at 1 h before LPS was administered, and then blood samples were obtained from the cheek of each mouse 1 h after LPS injection. The TNFα levels of the plasma samples were significantly high in the LPS-treated groups with/without C87 and GSK872, but there was no difference between mice pretreated with C87 or GSK872 and vehicle-treated mice following LPS injection. Data are presented as mean ± SEM; n = 7 per group. One-way ANOVA; Bonferroni post hoc test vs. vehicle- and LPS-treated mice; ***p < 0.001. [file 12974_2022_2406_MOESM1_ESM.tif]

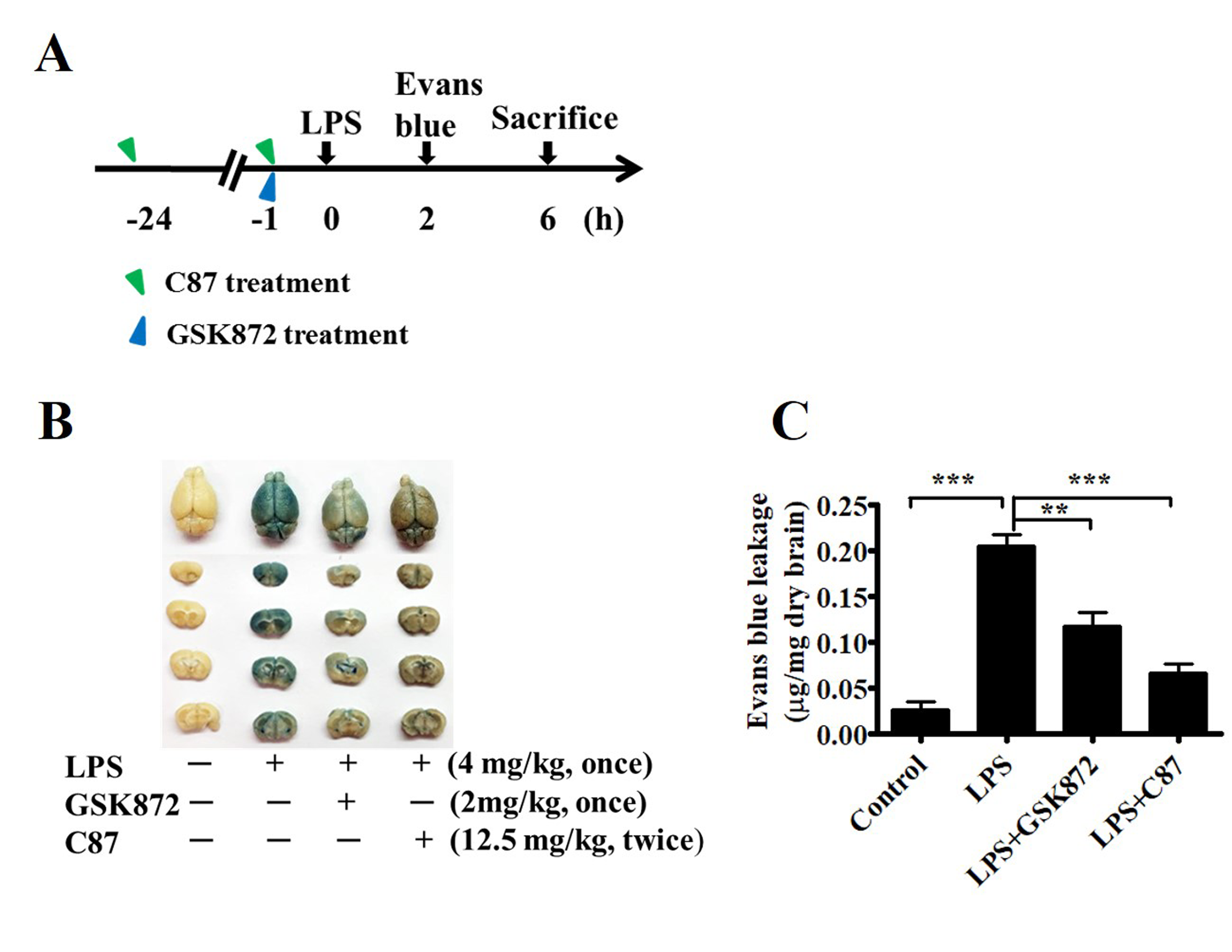

Supplement: Supplementary file 2 — Additional file 2: Figure S2. Effects of GSK872 and C87 pretreatment on brain vascular permeability in LPS-treated mice (A) The experimental protocol. The mice were injected with 3% Evans blue dye (i.p.) 2 h after vehicle (saline) and LPS (4 mg/kg, i.p.) was administrated, and the brains were obtained 4 h later. (B) Representative photographs of brains and coronal brain sections with Evans blue extravasation. Evans Blue leakage into brain was measured by spectrophotometer at 620 nm and quantified according to a standard curve. The results were presented as µg of Evans Blue per mg of dried brain tissue. (C) The density levels of Evans blue leakage in hippocampus was higher in LPS-treated mice, which was significantly reduced in C87 and GSK872 pretreated mice (n = 3 per group). Data represent the mean ± SEM of values. One way ANOVA; ***p < 0.001; **p < 0.01. [file 12974_2022_2406_MOESM2_ESM.tif]
